# Supplementary material for: Simultaneous estimation of gene regulatory network structure and RNA kinetics from single cell gene expression
Source: bioRxiv. 2023 Sep 23:2023.09.21.558277. Preprint. [Version 1] doi: 10.1101/2023.09.21.558277 (PMC10542544; doi:10.1101/2023.09.21.558277)
Supplement: Supplement 11 [file NIHPP2023.09.21.558277v1-supplement-11.pdf]

bioRxiv preprint doi: https://doi.org/10.1101/2023.09.21.558277; this version posted September 23, 2023. The copyright holder for this preprint (which was not certified by peer review) is the author/funder, who has granted bioRxiv a license to display the preprint in perpetuity. It is made available under aCC-BY 4.0 International license.

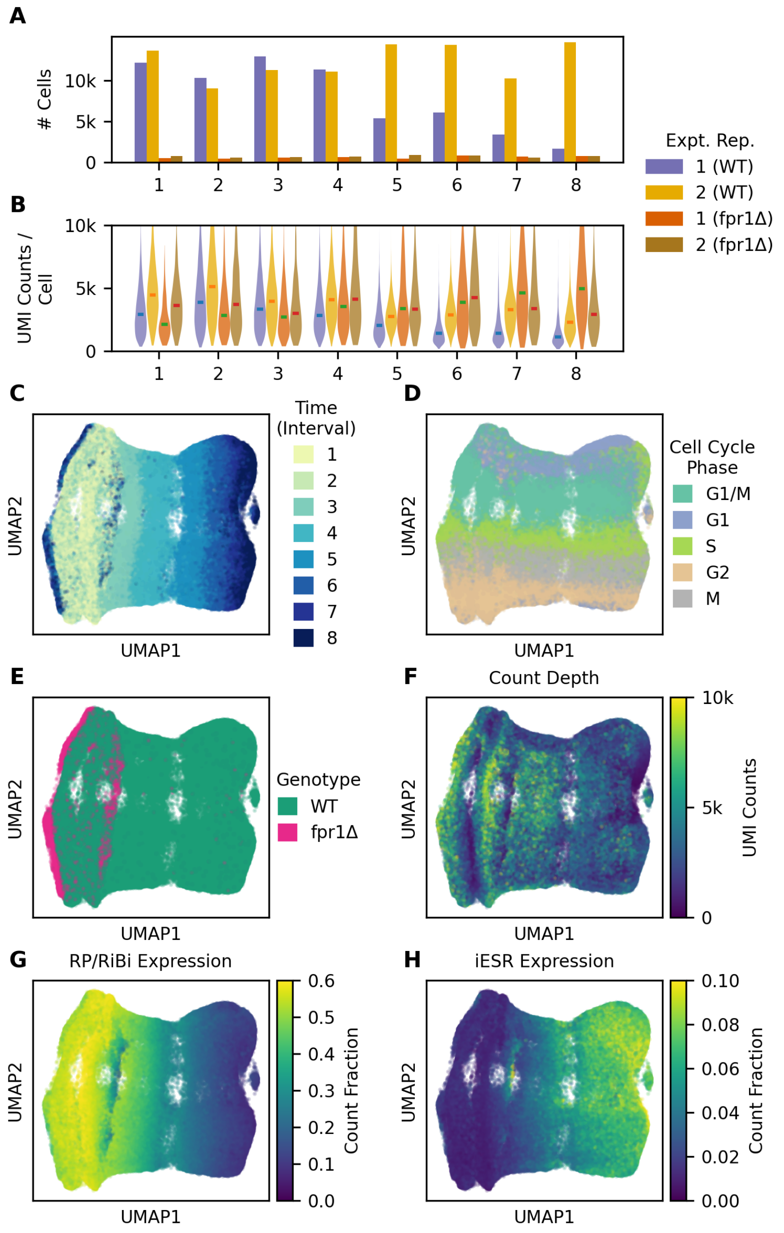

Supplemental Figure 1: Summary of *Saccharomyces cerevisiae* single-cell transcriptomics (**A**) Number of wild-type (WT) and rapamycin non-responsive (fpr1Δ) cells captured in each sampling interval for each experimental replicate. (**B**) Distribution of the number of counts after processing Unique Molecular Identifiers (UMIs) in each sampling interval, experimental replicate, and yeast genotype. (**C**) 2D UMAP plot of single-cell *Saccharomyces cerevisiae* responding to rapamycin treatment, colored by sampling time, reproduced from Figure 1C. (**D-H**) 2D UMAP plot colored by estimated cell-cycle phase (**D**), yeast genotype (**E**), number of counts per cell (**F**), Ribosomal protein (RP) and Ribosomal biogenesis (RiBi) gene expression as a proportion of total gene expression (**G**), and Induced Environmental Stress Response (iESR) gene expression as a proportion of total gene expression (**H**).

bioRxiv preprint doi: https://doi.org/10.1101/2023.09.21.558277; this version posted September 23, 2023. The copyright holder for this preprint (which was not certified by peer review) is the author/funder, who has granted bioRxiv a license to display the preprint in perpetuity. It is made available under aCC-BY 4.0 International license.

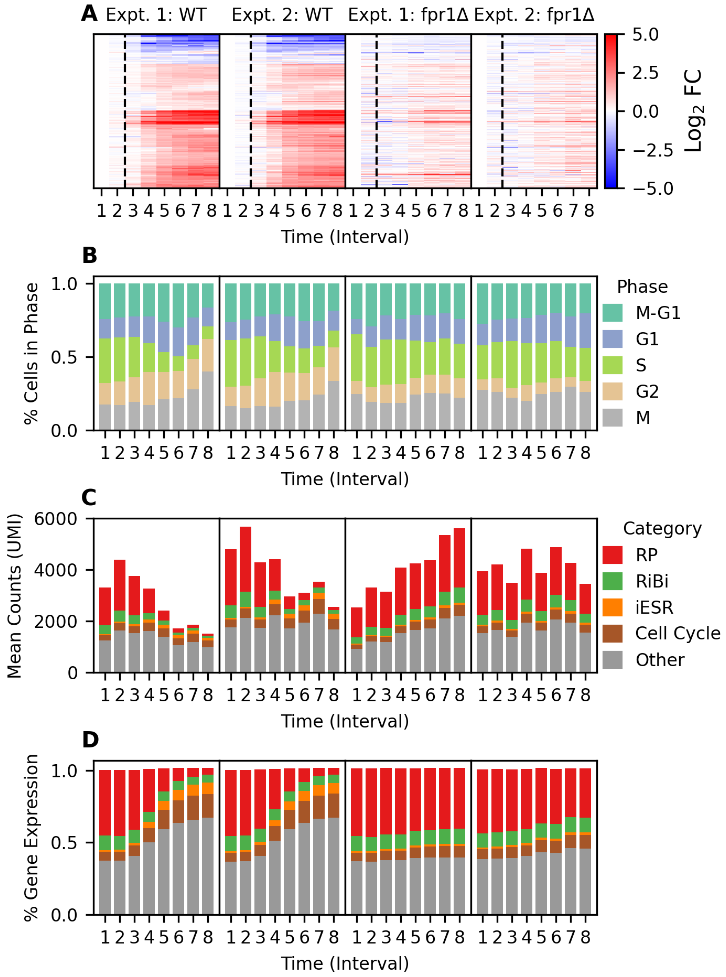

Supplemental Figure 2: Summary of *Saccharomyces cerevisiae* single-cell changes between sampling intervals and experimental replicates (**A**) Differential expression (Log_2 Fold Change compared to untreated sampling interval 1 of yeast transcriptome in wild-type (WT) and rapamycin non-responsive (fpr1Δ) cells in each experimental replicate. (**B**) Proportion of the number of cells in each estimated cell-cycle phase between sampling intervals and experimental replicates. (**C**) Median proportion of the number of counts from each category of genes between sampling intervals and experimental replicates. (**D**) Median absolute number of counts from each category of genes between sampling intervals and experimental replicates.

bioRxiv preprint doi: https://doi.org/10.1101/2023.09.21.558277; this version posted September 23, 2023. The copyright holder for this preprint (which was not certified by peer review) is the author/funder, who has granted bioRxiv a license to display the preprint in perpetuity. It is made available under aCC-BY 4.0 International license.

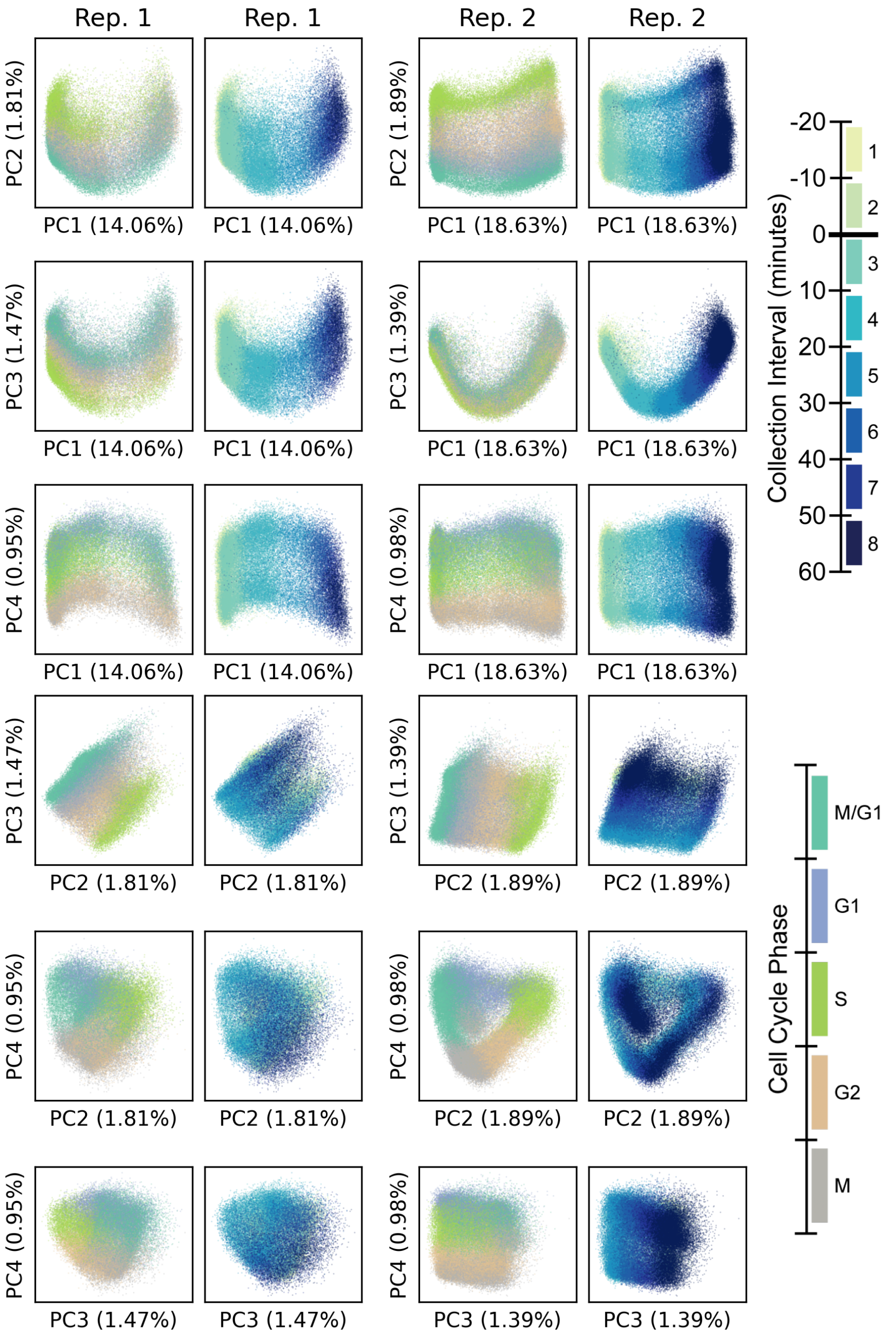

Supplemental Figure 3: Principal component (PC) plots of the first four principal components (PC1-PC4), annotated with percent variance explained by that PC. Cells from different experimental replicates are plotted separately. Only wild-type cells are plotted. Each plot is duplicated and colored by cell cycle phase (**left**) or sampling interval (**right**).

bioRxiv preprint doi: https://doi.org/10.1101/2023.09.21.558277; this version posted September 23, 2023. The copyright holder for this preprint (which was not certified by peer review) is the author/funder, who has granted bioRxiv a license to display the preprint in perpetuity. It is made available under aCC-BY 4.0 International license.

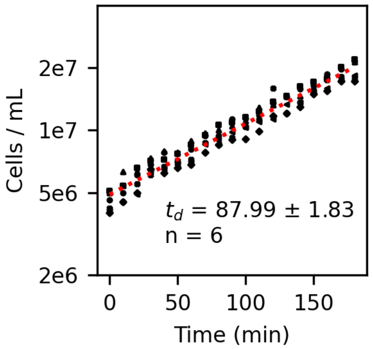

Supplemental Figure 4: Growth rate of exponentially growing FY4/5 cells in rich YPD media. Individual cell densities for 6 replicate cultures plotted against time. Dotted line is the ordinary least-squares regression line of the form $log_2(Density) = at + b$. Doubling time t_d (min) is $^1/_a$ of the regression slope $\pm$ standard error.

bioRxiv preprint doi: https://doi.org/10.1101/2023.09.21.558277; this version posted September 23, 2023. The copyright holder for this preprint (which was not certified by peer review) is the author/funder, who has granted bioRxiv a license to display the preprint in perpetuity. It is made available under aCC-BY 4.0 International license.

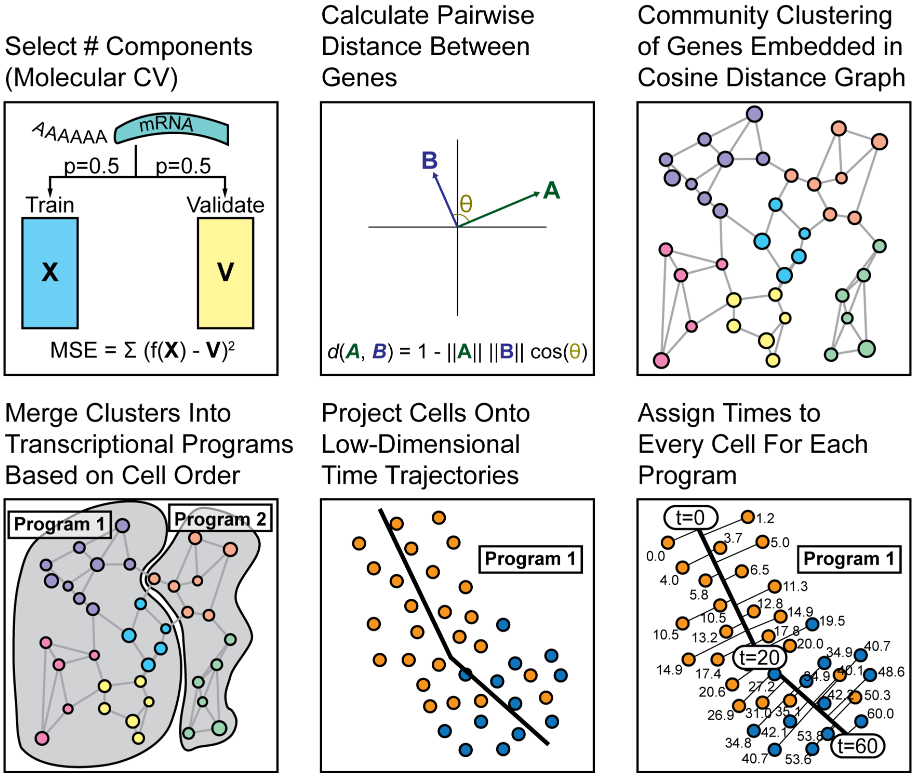

Supplemental Figure 5: Schematic diagram of computational method for assigning cells specific times in minutes for response to rapamycin treatment and the cell cycle. **1**: Expression data is denoised by retaining the first n PCs, chosen by molecular cross validation, and discarding variance from other PCs. **2**: Genes are embedded into a graph using Cosine Distance as the distance metric. **3**: Genes are grouped into clusters based on the Leiden community clustering algorithm. **4**: Clusters of genes are further merged into programs based on circular rank correlation between the first principal component, calculated separately for each gene cluster. **5**: Low-dimensional time trajectories are constructed by splines between the centroids of metadata-assigned cell groups in m dimensional principal component space, where m is chosen for each program's genes separately by molecular cross validation. **6**: Times in minutes are assigned by projecting cells onto the low-dimensional time trajectories.

bioRxiv preprint doi: https://doi.org/10.1101/2023.09.21.558277; this version posted September 23, 2023. The copyright holder for this preprint (which was not certified by peer review) is the author/funder, who has granted bioRxiv a license to display the preprint in perpetuity. It is made available under aCC-BY 4.0 International license.

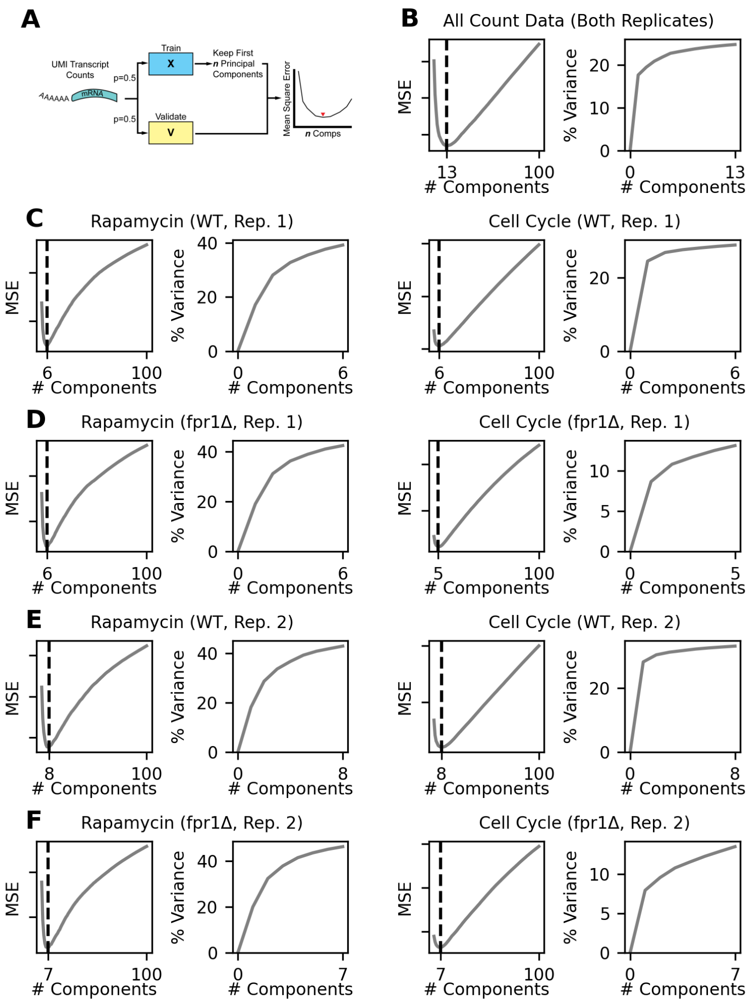

Supplemental Figure 6: Molecular cross-validation (MCV) to select the number of principal components for gene to program assignment (**A**) MCV schematic. UMI counts are randomly partitioned into two cells by genes matrices, one of which is used for testing (X) and one of which is used for validation. Test matrix is denoised by projecting into principal component space, retaining only the top n principal components, and then transforming back into expression space. The optimal number of principal components minimizes mean squared error between the denoised test matrix X and the unmodified validation matrix V. (**B**) MCV results for all cells and all genes, showing mean squared error (MSE) that identifies 13 PCs as optimal, and the cumulative variance explained by 13 PCs. (**C-F**) MCV results for each program (rapaycin treatment and cell cycle) shown separately for replicate 1 wild-type (**C**), replicate 1 fpr1Δ (**D**), replicate 2 wild-type (**E**), and replicate 2 fpr1Δ (**F**).

bioRxiv preprint doi: https://doi.org/10.1101/2023.09.21.558277; this version posted September 23, 2023. The copyright holder for this preprint (which was not certified by peer review) is the author/funder, who has granted bioRxiv a license to display the preprint in perpetuity. It is made available under aCC-BY 4.0 International license.

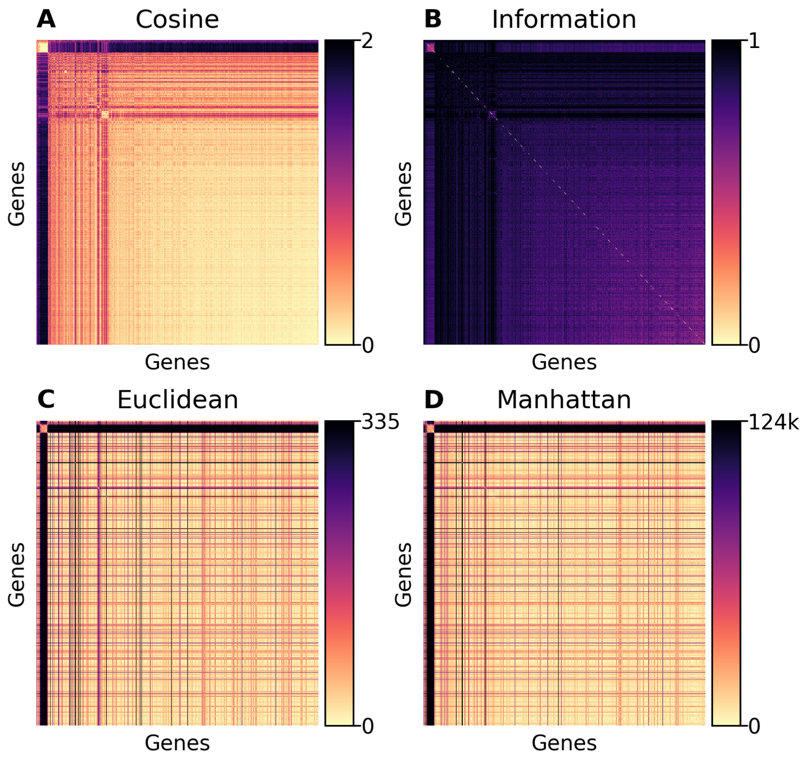

Supplemental Figure 7: Distance metric heatmaps between genes for all cells for (**A**) Cosine distance, (**B**) Information distance, (**C**) Euclidean distance, and (**D**) Manhattan distance. Genes are ordered identically for each panel, based on hierarchical clustering using cosine distance.

bioRxiv preprint doi: https://doi.org/10.1101/2023.09.21.558277; this version posted September 23, 2023. The copyright holder for this preprint (which was not certified by peer review) is the author/funder, who has granted bioRxiv a license to display the preprint in perpetuity. It is made available under aCC-BY 4.0 International license.

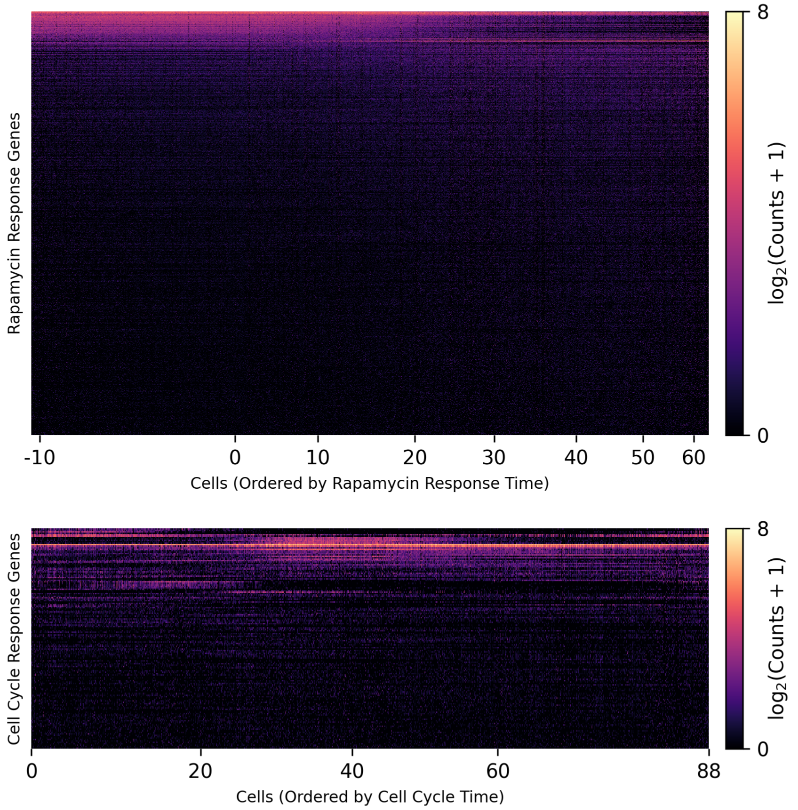

Supplemental Figure 8: Expression heatmaps of genes for each program on the Y-axis with wild-type cells (n=162,529) on the X-axis (**A**) Expression of rapamycin treatment response genes (n=5379), with cells on the X-axis ordered by rapamycin response time (**B**) Expression of cell cycle response genes (n=348), with cells on the X-axis ordered by cell cycle time

bioRxiv preprint doi: https://doi.org/10.1101/2023.09.21.558277; this version posted September 23, 2023. The copyright holder for this preprint (which was not certified by peer review) is the author/funder, who has granted bioRxiv a license to display the preprint in perpetuity. It is made available under aCC-BY 4.0 International license.

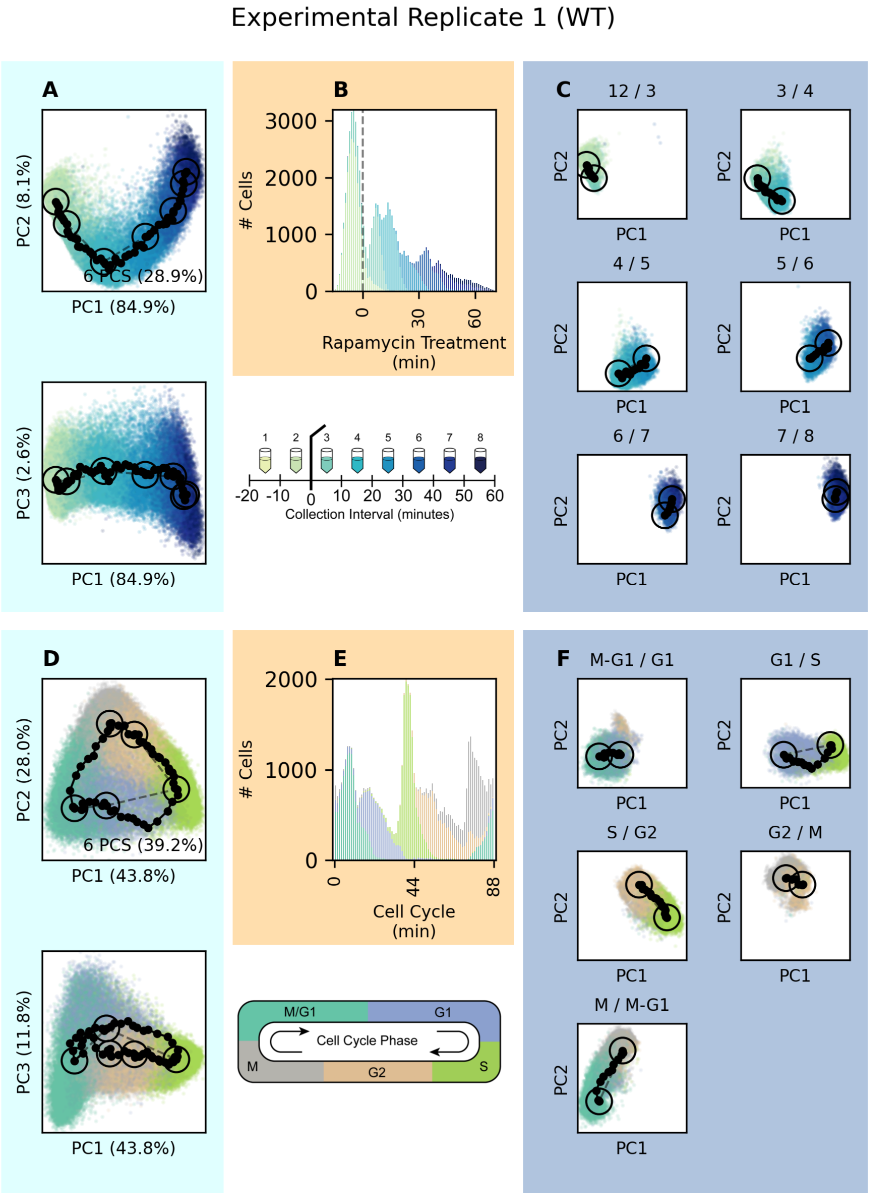

Supplemental Figure 9: Assigning cell times to experimental replicate 1 wild-type cells. (**A**) Principal component plots of cells using only rapamycin treatment program genes. Collection time centroids are circled and connected by dashed spline. 6 PCs are used in total for time projection. (**B**) Histogram of assigned cell cycle times, colored by collection interval. (**C**) Principal component plots of cells in adjacent collection intervals, showing their centroids circled, the spline connection as a dashed line, and the shortest-walk path between them. (**D**) Principal component plots of cells using only cell cycle program genes. Cell cycle phase centroids are circled and connected by dashed spline. 6 PCs are used in total for time projection. (**E**) Histogram of assigned cell cycle times, colored by cell cycle phase. (**F**) Principal component plots of cells in adjacent phases, showing their centroids circled, the spline connection as a dashed line, and the shortest-walk path between them.

bioRxiv preprint doi: https://doi.org/10.1101/2023.09.21.558277; this version posted September 23, 2023. The copyright holder for this preprint (which was not certified by peer review) is the author/funder, who has granted bioRxiv a license to display the preprint in perpetuity. It is made available under aCC-BY 4.0 International license.

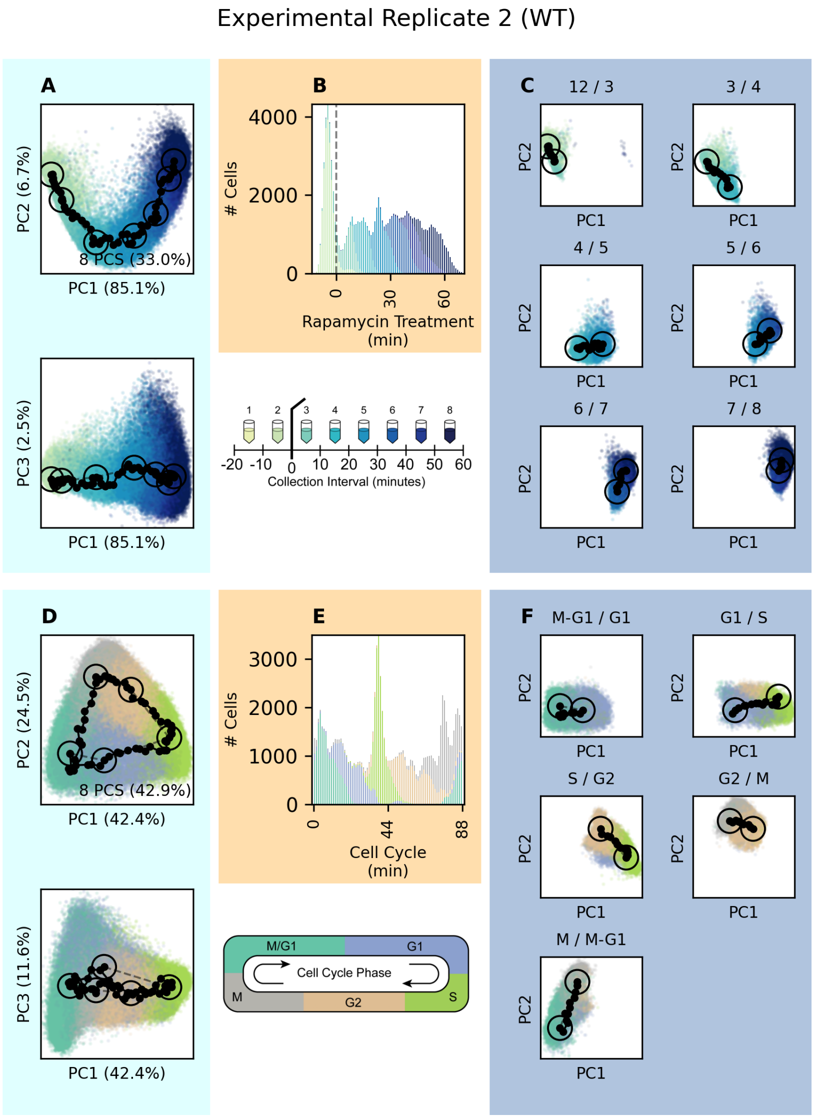

Supplemental Figure 10: Assigning cell times to experimental replicate 2 wild-type cells. (**A**) Principal component plots of cells using only rapamycin treatment program genes. Collection time centroids are circled and connected by dashed spline. 8 PCs are used in total for time projection. (**B**) Histogram of assigned cell cycle times, colored by collection interval. (**C**) Principal component plots of cells in adjacent collection intervals, showing their centroids circled, the spline connection as a dashed line, and the shortest-walk path between them. (**D**) Principal component plots of cells using only cell cycle program genes. Cell cycle phase centroids are circled and connected by dashed spline. 8 PCs are used in total for time projection. (**E**) Histogram of assigned cell cycle times, colored by cell cycle phase. (**F**) Principal component plots of cells in adjacent phases, showing their centroids circled, the spline connection as a dashed line, and the shortest-walk path between them.

bioRxiv preprint doi: https://doi.org/10.1101/2023.09.21.558277; this version posted September 23, 2023. The copyright holder for this preprint (which was not certified by peer review) is the author/funder, who has granted bioRxiv a license to display the preprint in perpetuity. It is made available under aCC-BY 4.0 International license.

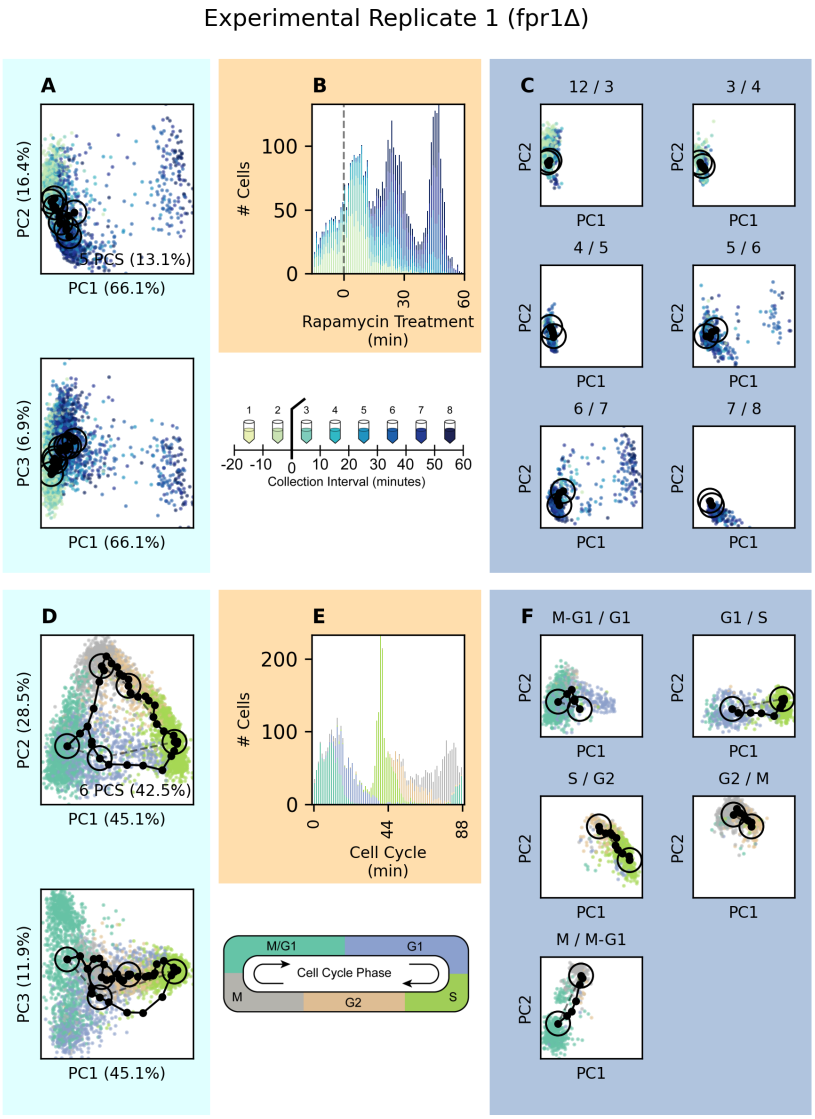

Supplemental Figure 11: Assigning cell times to experimental replicate 1 fpr1Δ cells. (**A**) Principal component plots of cells using only rapamycin treatment program genes. Collection time centroids are circled and connected by dashed spline. 5 PCs are used in total for time projection. (**B**) Histogram of assigned cell cycle times, colored by collection interval. (**C**) Principal component plots of cells in adjacent collection intervals, showing their centroids circled, the spline connection as a dashed line, and the shortest-walk path between them. (**D**) Principal component plots of cells using only cell cycle program genes. Cell cycle phase centroids are circled and connected by dashed spline. 6 PCs are used in total for time projection. (**E**) Histogram of assigned cell cycle times, colored by cell cycle phase. (**F**) Principal component plots of cells in adjacent phases, showing their centroids circled, the spline connection as a dashed line, and the shortest-walk path between them.

bioRxiv preprint doi: https://doi.org/10.1101/2023.09.21.558277; this version posted September 23, 2023. The copyright holder for this preprint (which was not certified by peer review) is the author/funder, who has granted bioRxiv a license to display the preprint in perpetuity. It is made available under aCC-BY 4.0 International license.

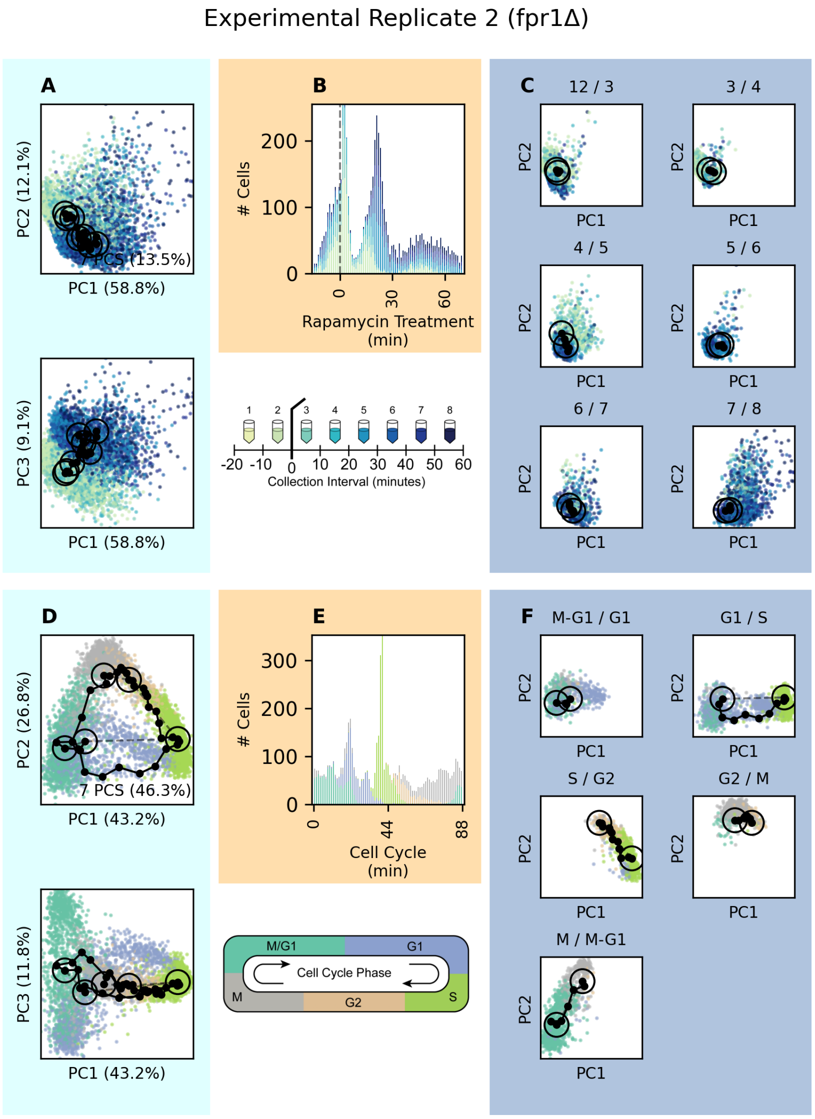

Supplemental Figure 12: Assigning cell times to experimental replicate 2 fpr1Δ cells. (**A**) Principal component plots of cells using only rapamycin treatment program genes. Collection time centroids are circled and connected by dashed spline. 7 PCs are used in total for time projection. (**B**) Histogram of assigned cell cycle times, colored by collection interval. (**C**) Principal component plots of cells in adjacent collection intervals, showing their centroids circled, the spline connection as a dashed line, and the shortest-walk path between them. (**D**) Principal component plots of cells using only cell cycle program genes. Cell cycle phase centroids are circled and connected by dashed spline. 7 PCs are used in total for time projection. **E**) Histogram of assigned cell cycle times, colored by cell cycle phase. (**F**) Principal component plots of cells in adjacent phases, showing their centroids circled, the spline connection as a dashed line, and the shortest-walk path between them.

bioRxiv preprint doi: https://doi.org/10.1101/2023.09.21.558277; this version posted September 23, 2023. The copyright holder for this preprint (which was not certified by peer review) is the author/funder, who has granted bioRxiv a license to display the preprint in perpetuity. It is made available under aCC-BY 4.0 International license.

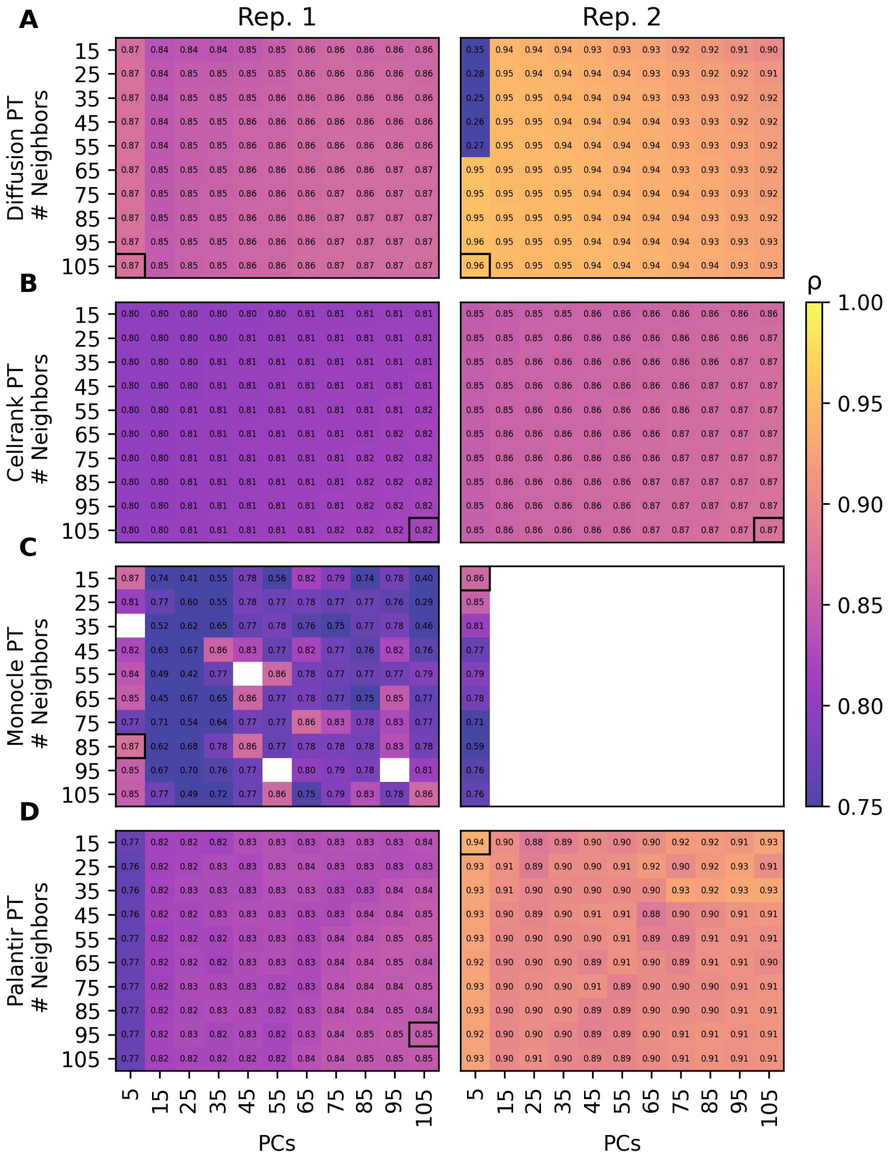

Supplemental Figure 13: Grid search to identify optimal pseudotime *k*-nearest neighbors (Y-axis) and principal component (X-axis) hyperparameters. Scores are Spearman rho coefficients between pseudotime and the sampling interval time labels. White-background grid squares with no score indicate the presence of non-finite pseudotime values for some cells that cannot be scored. (**A-D**) Scores are reported by experimental replicate for only wild-type cells. Methods are (**A**) scanpy diffusion pseudotime where the number of diffusion components is set equal to the number of principal components, (**B**) cellrank CytoTrace pseudotime, (**C**) monocle3 pseudotime, and (**D**) palantir pseudotime where the number of diffusion components is set equal to the number of principal components.

bioRxiv preprint doi: https://doi.org/10.1101/2023.09.21.558277; this version posted September 23, 2023. The copyright holder for this preprint (which was not certified by peer review) is the author/funder, who has granted bioRxiv a license to display the preprint in perpetuity. It is made available under aCC-BY 4.0 International license.

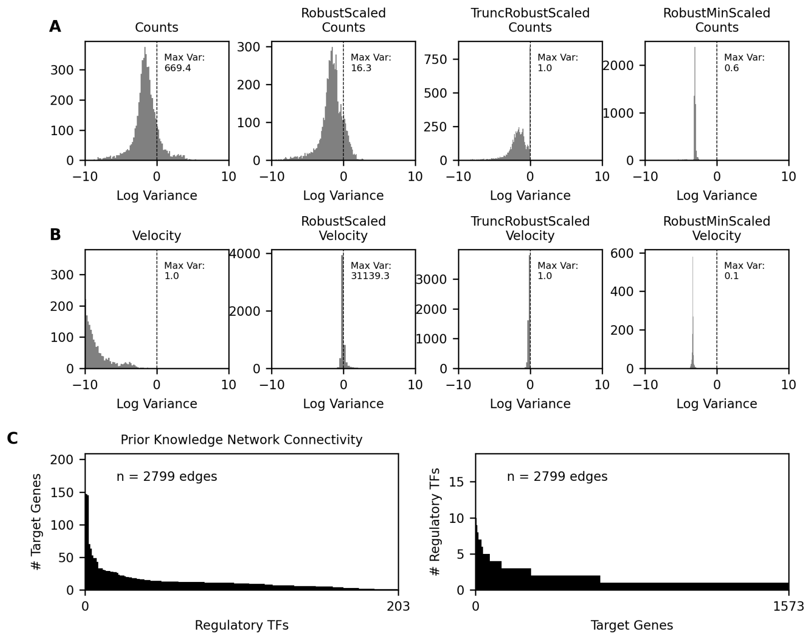

Supplemental Figure 14: Comparison of Preprocessing and Prior Network Structure (**A**) Histograms of variance per gene in RNA expression (count) data when unscaled, when scaled to interquartile range (RobustScaled), when scaled to interquartile range with a maximum gene variance of 1 (TruncRobustScaled), and when scaled to values between 0-1 (RobustMinScaled). The maximum variance for a feature is annotated on each plot. (**B**) Histograms of variance per gene in RNA velocity (rate of change) as in **A** (**C**) Connectivity histogram of prior network knowledge between 1573 genes and 203 regulatory TFs, with 2799 regulatory network edges

bioRxiv preprint doi: https://doi.org/10.1101/2023.09.21.558277; this version posted September 23, 2023. The copyright holder for this preprint (which was not certified by peer review) is the author/funder, who has granted bioRxiv a license to display the preprint in perpetuity. It is made available under aCC-BY 4.0 International license.

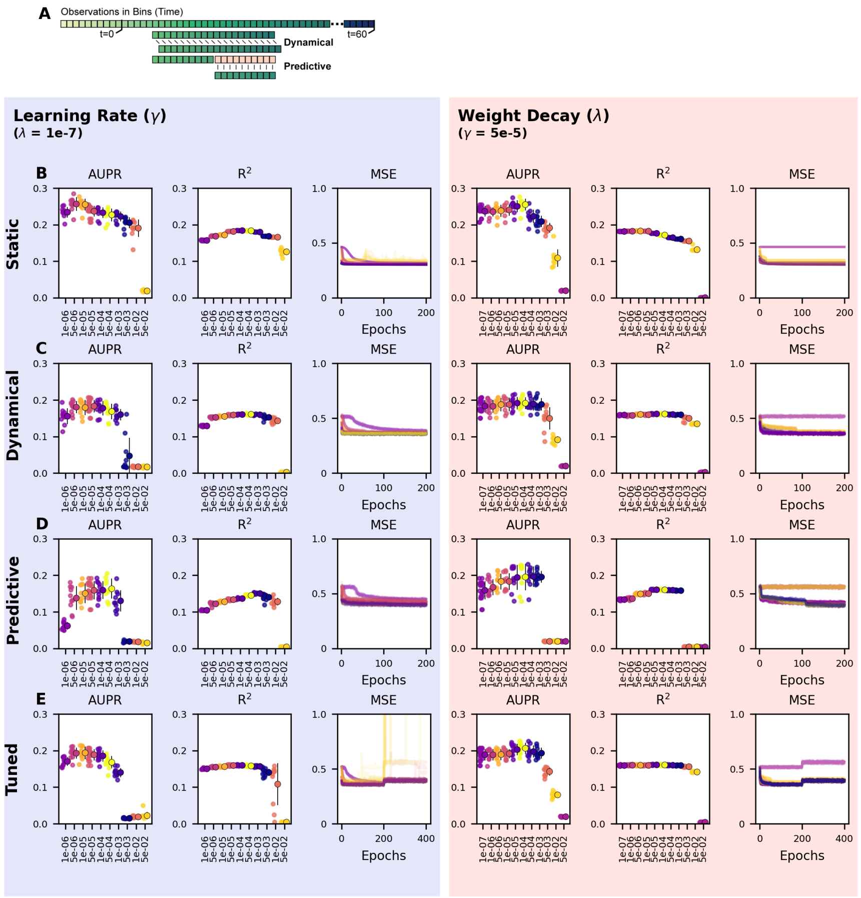

Supplemental Figure 15: Hyperparameter search for rapamycin response regulatory network inference models. Individual cells are binned into 70 one-minute-wide bins between -10 minutes and 60 minutes, and 20 minute cell trajectories are randomly selected for each training epoch by choosing one cell from each bin. (**A**) Comparison of training strategies for Dynamical models (comparing $t + 1$ predictions only) and Predictive models (comparing a sequence of 10 predictions) (**B**) Static model performance quantified by Area Under the Precision Recall curve (AUPR), coefficient of determination (R^2), and mean squared error (MSE) per training epoch. Performance is shown for a range of Adam optimizer learning rates (γ) with weight decay (λ) held constant, and a range of weight decays with learning rate held constant. (**C**) Dynamical model ($t + 1$ predictions) performance quantified as in **B** (**D**) Predictive model (10 sequential predictions) performance quantified as in **B** (**E**) Tuned model (training 200 epochs with the dynamical training strategy, followed by 200 epochs with the predictive model training strategy) performance quantified as in **B**

bioRxiv preprint doi: https://doi.org/10.1101/2023.09.21.558277; this version posted September 23, 2023. The copyright holder for this preprint (which was not certified by peer review) is the author/funder, who has granted bioRxiv a license to display the preprint in perpetuity. It is made available under aCC-BY 4.0 International license.

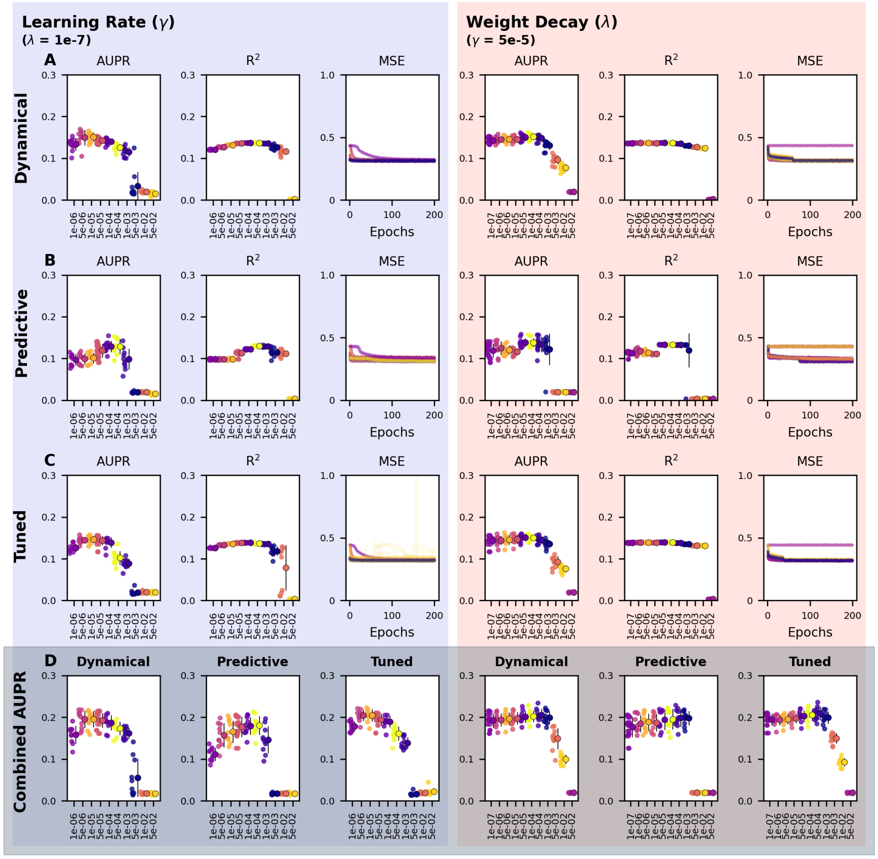

Supplemental Figure 16: Hyperparameter search for cell cycle regulatory network inference models. Individual cells are binned into 88 one-minute-wide bins between 0 minutes and 88 minutes, and 20 minute cell trajectories are randomly selected for each training epoch by choosing one cell from each bin. (**A**) Dynamical model ($t + 1$ predictions) performance quantified by AUPR, R^2, and mean squared error (MSE) per training epoch. Performance is shown for a range of Adam optimizer learning rates (γ) with weight decay (λ) held constant, and a range of weight decays with learning rate held constant. (**B**) Predictive model (10 sequential predictions) performance quantified as in **A** (**C**) Tuned model (training 200 epochs with the dynamical training strategy, followed by 200 epochs with the predictive model training strategy) performance quantified as in **A** (**D**) GRNs combined from rapamycin and cell cycle response models by taking the maximum explained relative variance for all regulatory edges from each model. Performance is evaluated by AUPR.

bioRxiv preprint doi: https://doi.org/10.1101/2023.09.21.558277; this version posted September 23, 2023. The copyright holder for this preprint (which was not certified by peer review) is the author/funder, who has granted bioRxiv a license to display the preprint in perpetuity. It is made available under aCC-BY 4.0 International license.

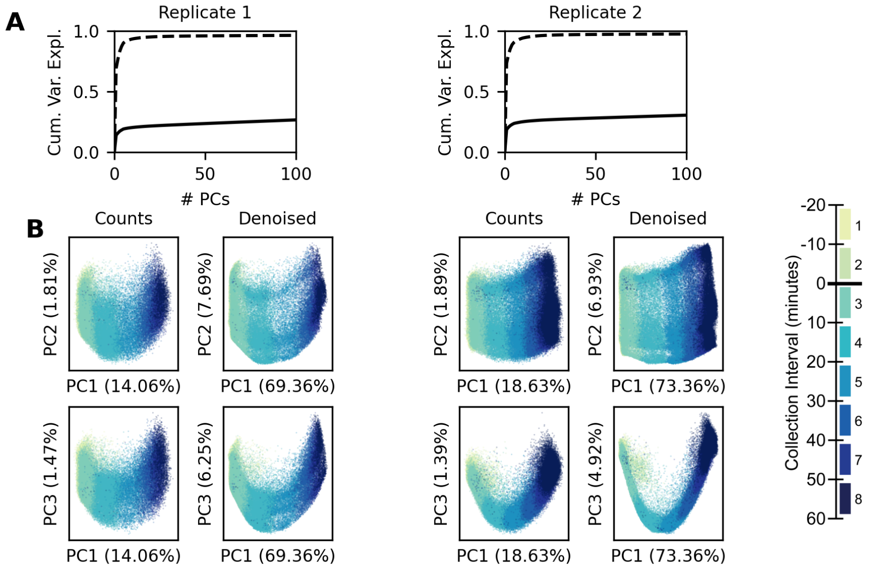

Supplemental Figure 17: Denoising count data (**A**) Cumulative variance explained per principal component for raw count data (solid line) and denoised count data (dashed line), plotted separately for each experimental replicate (**B**) Principal components PC1 & PC2 and PC1 & PC3 plotted against each other for raw count data and denoised count data. Experimental replicates are plotted separately. Individual cells are colored by collection interval.

bioRxiv preprint doi: https://doi.org/10.1101/2023.09.21.558277; this version posted September 23, 2023. The copyright holder for this preprint (which was not certified by peer review) is the author/funder, who has granted bioRxiv a license to display the preprint in perpetuity. It is made available under aCC-BY 4.0 International license.

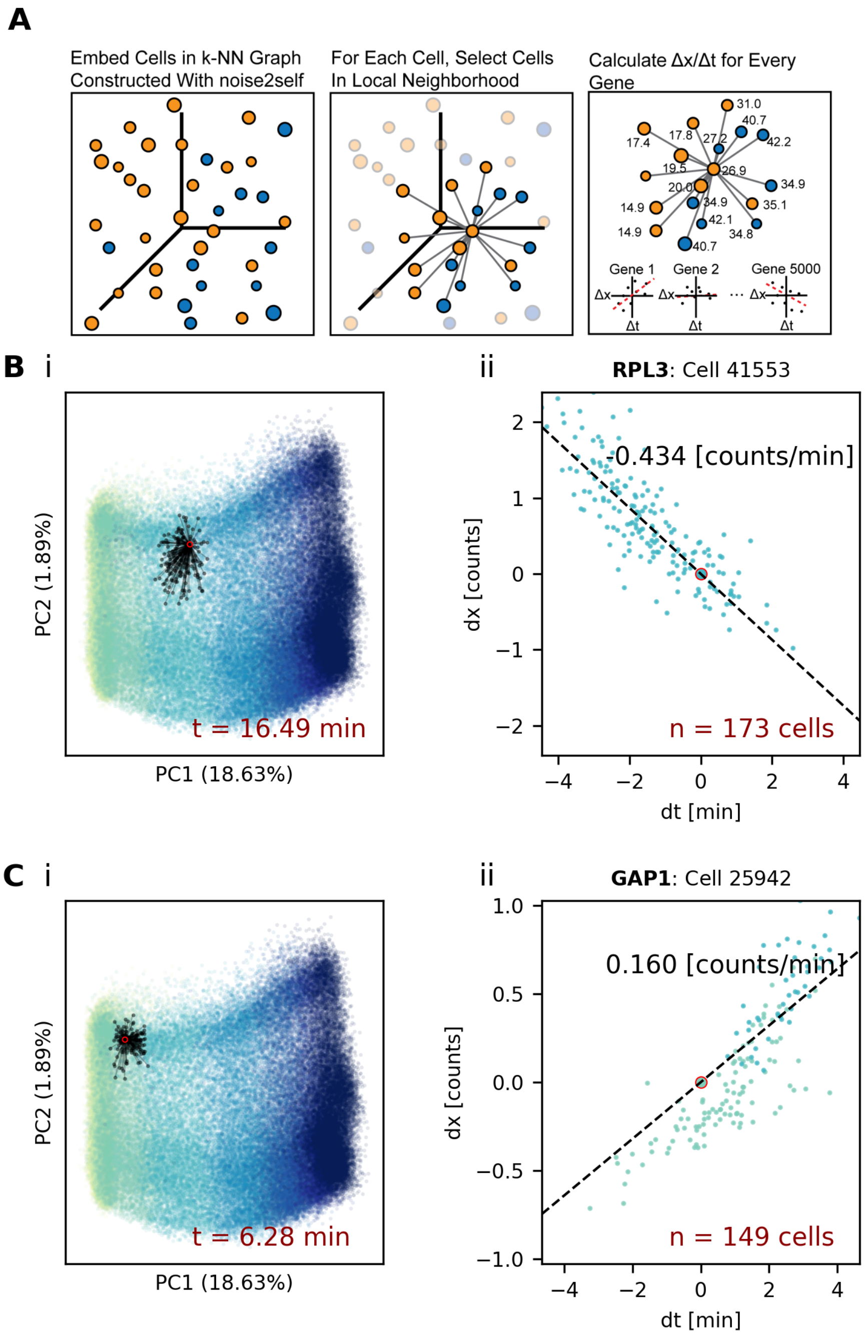

Supplemental Figure 18: Calculation of RNA velocity (rate of change) (**A**) Schematic showing *k*-NN network construction and RNA velocity regression within local neighborhood for each cell. (**B**) RNA velocity regression for ***RPL3*** at a randomly chosen cell, highlighted on the PC plot (i) and showing only cells connected in the *k*-NN network for velocity regression. (**C**) RNA velocity regression for ***GAP1*** at a randomly chosen cell as in **B**.

bioRxiv preprint doi: https://doi.org/10.1101/2023.09.21.558277; this version posted September 23, 2023. The copyright holder for this preprint (which was not certified by peer review) is the author/funder, who has granted bioRxiv a license to display the preprint in perpetuity. It is made available under aCC-BY 4.0 International license.

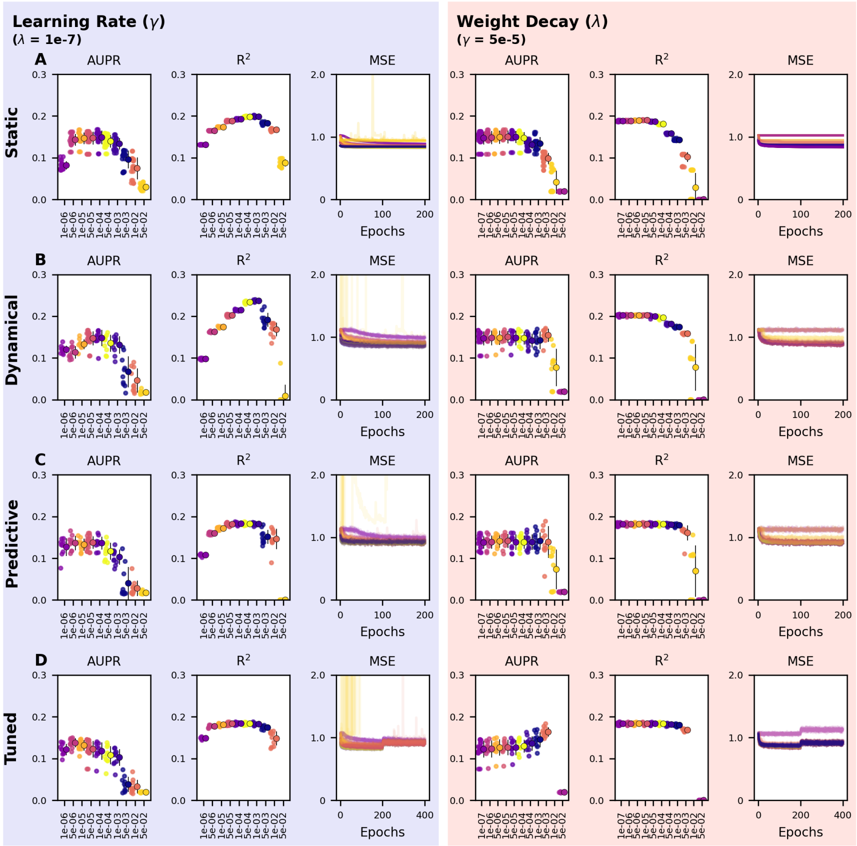

Supplemental Figure 19: Hyperparameter search for rapamycin velocity inference models, with cells binned as in Supplemental Figure 15 Models are trained with counts as input and RNA velocity as output. (**A**) Static model performance quantified by Area Under the Precision Recall curve (AUPR), coefficient of determination (R^2), and mean squared error (MSE) per training epoch. Performance is shown for a range of Adam optimizer learning rates (γ) with weight decay (λ) held constant, and a range of weight decays with learning rate held constant. (**B**) Dynamical model ($t + 1$ prediction) performance quantified as in **A** (**C**) Predictive model (10 sequential predictions) performance quantified as in **A**. Model input at $t + 1$ is counts t plus velocity t. (**D**) Tuned model (training 200 epochs with the dynamical training strategy, followed by 200 epochs with the predictive model training strategy) performance quantified as in **A**

bioRxiv preprint doi: https://doi.org/10.1101/2023.09.21.558277; this version posted September 23, 2023. The copyright holder for this preprint (which was not certified by peer review) is the author/funder, who has granted bioRxiv a license to display the preprint in perpetuity. It is made available under aCC-BY 4.0 International license.

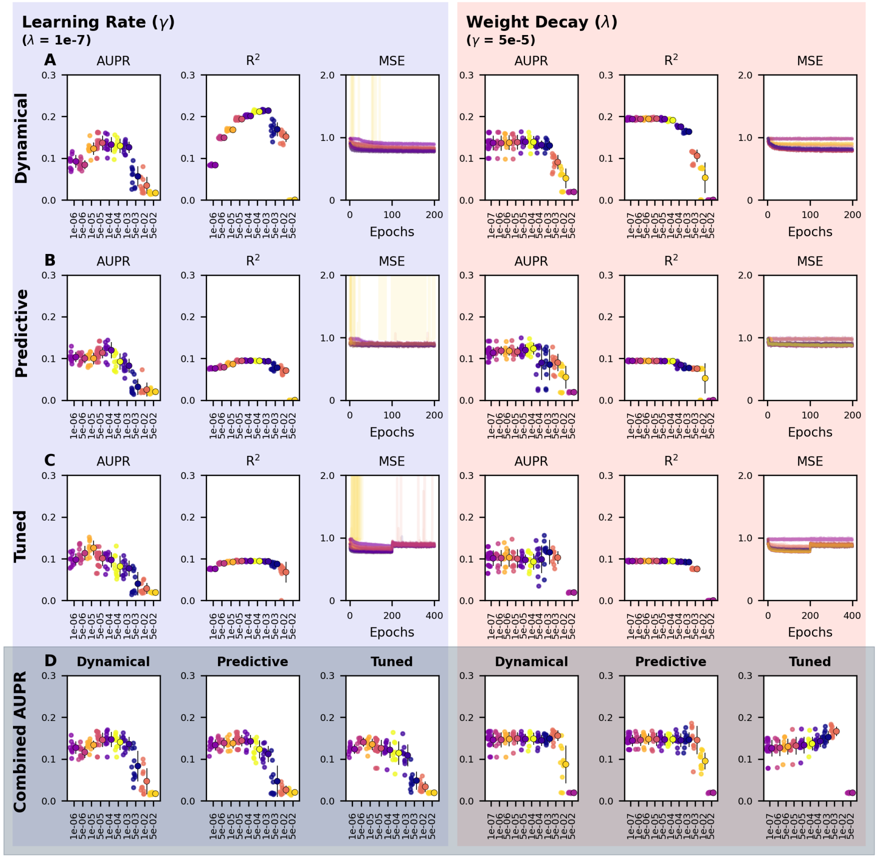

Supplemental Figure 20: Hyperparameter search for cell cycle velocity models, with cells binned as in Supplemental Figure 16 Models are trained with counts as input and RNA velocity as output. (**A**) Dynamical model ($t + 1$ prediction) performance quantified by Area Under the Precision Recall curve (AUPR), coefficient of determination (R^2), and mean squared error (MSE) per training epoch. Performance is shown for a range of Adam optimizer learning rates (γ) with weight decay (λ) held constant, and a range of weight decays with learning rate held constant. (**B**) Predictive model (10 sequential predictions) performance quantified as in **A**. Model input at $t + 1$ is counts t plus velocity t. (**C**) Tuned model (training 200 epochs with the dynamical training strategy, followed by 200 epochs with the predictive model training strategy) performance quantified as in **A**. (**D**) Regulatory networks combined from rapamycin and cell cycle velocity models by taking the maximum explained relative variance for all regulatory edges from each model. Performance is evaluated by AUPR.

bioRxiv preprint doi: https://doi.org/10.1101/2023.09.21.558277; this version posted September 23, 2023. The copyright holder for this preprint (which was not certified by peer review) is the author/funder, who has granted bioRxiv a license to display the preprint in perpetuity. It is made available under aCC-BY 4.0 International license.

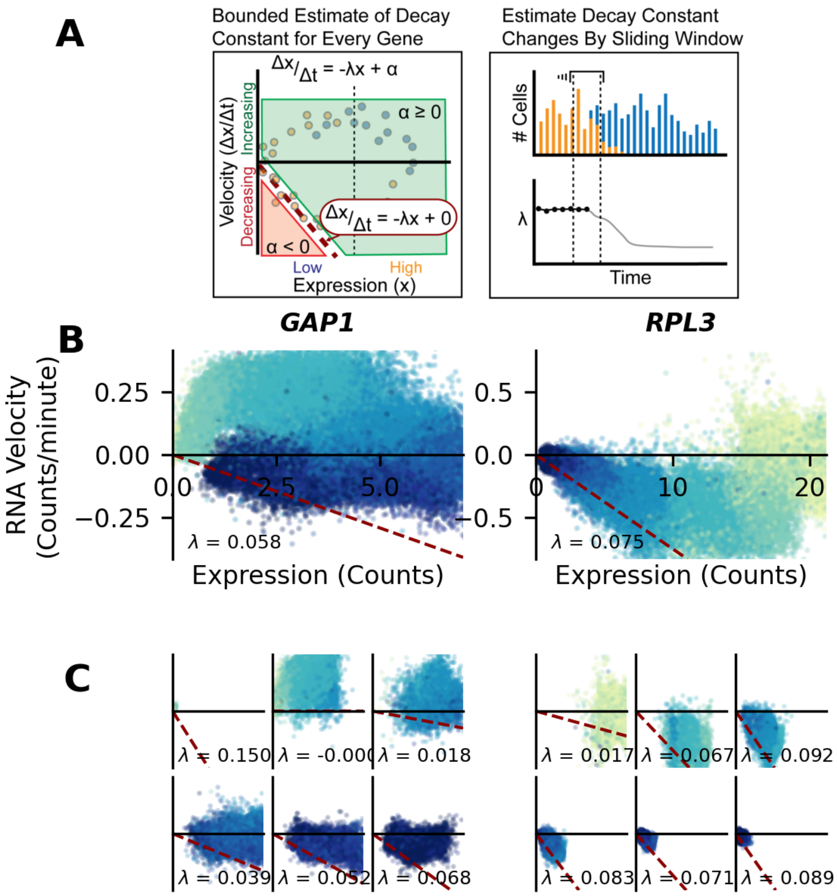

Supplemental Figure 21: Calculation of RNA decay rates as a dynamic, time-dependent parameter (**A**) Schematic showing bounded estimate of decay rates (**B**) Bounded estimate of *GAP1* and *RPL3* decay constant for all cells (**C**) Bounded estimate of decay rates from 10 minutes of cells (for each gene, -10 to 0, 0 to 10, 10 to 20 left to right in the top row; 20 to 30, 30 to 40, 40 to 50 left to right in the bottom row)

bioRxiv preprint doi: https://doi.org/10.1101/2023.09.21.558277; this version posted September 23, 2023. The copyright holder for this preprint (which was not certified by peer review) is the author/funder, who has granted bioRxiv a license to display the preprint in perpetuity. It is made available under aCC-BY 4.0 International license.

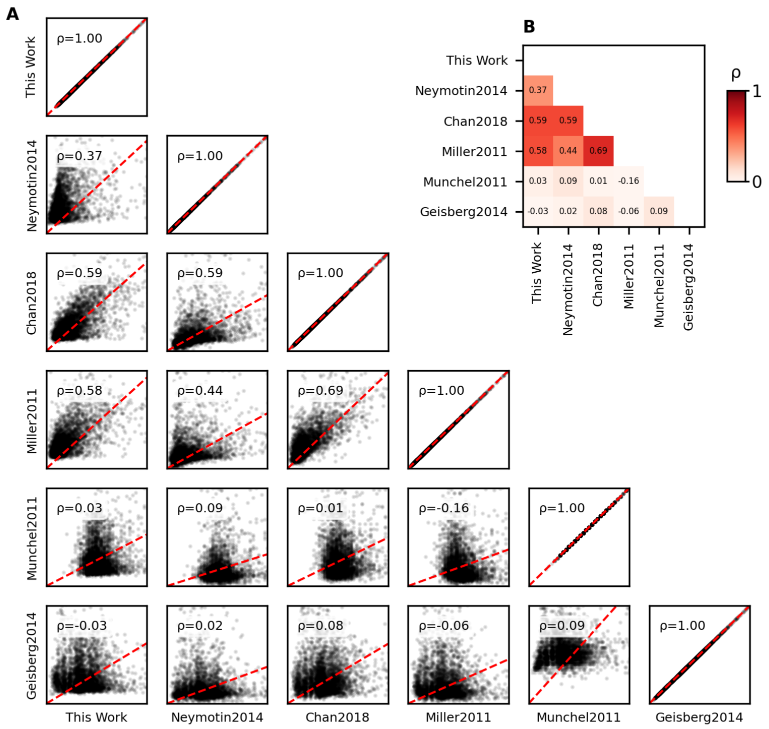

Supplemental Figure 22: Comparison of estimated mRNA half-lives prior to rapamycin treatment (This Work) with published mRNA half-lives measured by 4-thiouracil labeling in Neymotin2014 (*69*), Chan2018 (*70*), Munchel2011 (*36*), and Miller2011 (*71*). Also compared to decay rates measured by RNA polymerase anchor-away in Geisberg2014 (*72*). (**A**) Half-lives plotted for each data set. Dashed red line is linear regression with no intercept, and fit is reported as spearman correlation coefficient. (**B**) Heatmap of correlation coefficient from **A**.

bioRxiv preprint doi: https://doi.org/10.1101/2023.09.21.558277; this version posted September 23, 2023. The copyright holder for this preprint (which was not certified by peer review) is the author/funder, who has granted bioRxiv a license to display the preprint in perpetuity. It is made available under aCC-BY 4.0 International license.

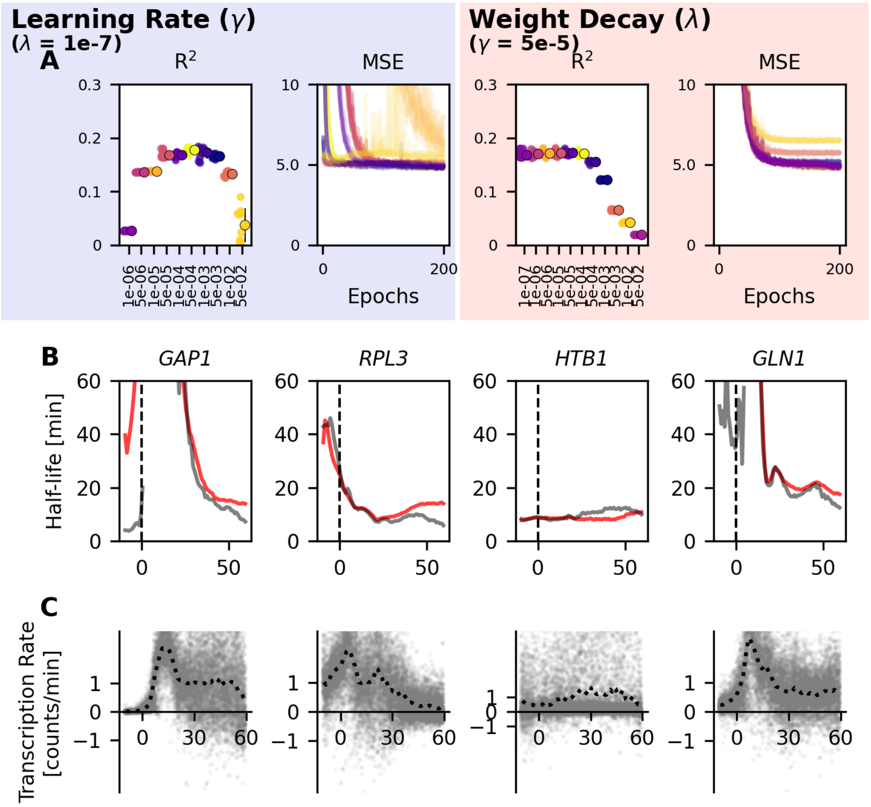

Supplemental Figure 23: Hyperparameter search for RNA decay model, with cells binned as in Supplemental Figure 15. Models are trained with counts as input and inferred decay rate as output. (**A**) Dynamical model $(t+1)$ performance quantified by coefficient of determination (R^2), and mean squared error (MSE) per training epoch. Performance is shown for a range of Adam optimizer learning rates (γ) with weight decay (λ) held constant, and a range of weight decays with learning rate held constant. (**B**) RNA half-life estimates from trained decay model for *GAP1*, *RPL3*, *HTB1*, and *GLN1* (red lines), compared to the RNA half-life estimates used as training data (gray). Training data RNA half-life estimates were calculated as in Supplemental Figure 21, using one-minute sliding windows. (**C**) RNA transcriptional rate estimates determined by subtracting estimated decay velocity ($-\lambda X$), with λ determined in **B**, from mRNA velocity

bioRxiv preprint doi: https://doi.org/10.1101/2023.09.21.558277; this version posted September 23, 2023. The copyright holder for this preprint (which was not certified by peer review) is the author/funder, who has granted bioRxiv a license to display the preprint in perpetuity. It is made available under aCC-BY 4.0 International license.

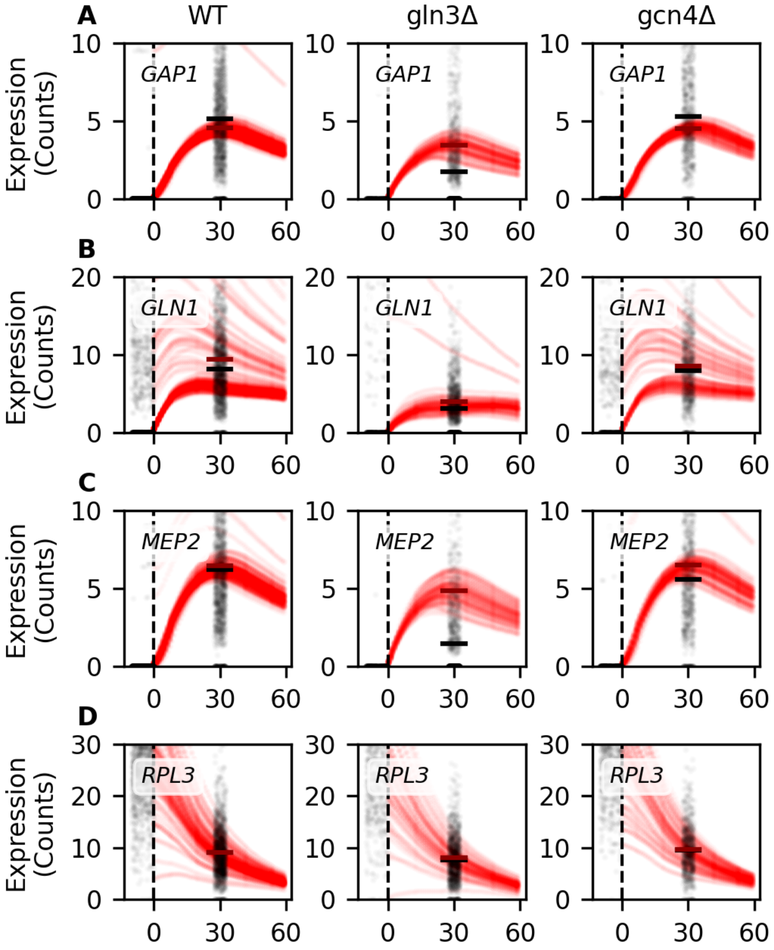

Supplemental Figure 24: Rapamycin response transcriptional predictions for cells with TFs deleted. *In silico* TF deletion sets TF activity to 0 at all times during prediction. (**A**) Model predictions for *GAP1* in an unperturbed wild-type model, a gln3Δ model, and a gcn4Δ model. Black points are observed counts from untreated (t=0 minutes) and treated (t=30 minutes) cells of the appropriate genotype, jittered for display. Red points are model-predicted counts. Thick black line at t=30 represents the mean of observed data. Thick red line at t=30 represents the mean of all predicted values between 28-32 minutes. (**B**) Model predictions for *GLN1*, plotted as in **A** (**C**) Model predictions for *MEP2*, plotted as in **A** (**D**) Model predictions for *RPL3*, plotted as in **A**
